# Effect of Fish Meal Substitution with Black Soldier Fly (*Hermetia illucens*) on Growth Performance, Feed Stability, Blood Biochemistry, and Liver and Gut Morphology of Siamese Fighting Fish (*Betta splendens*)

**DOI:** 10.1155/2023/6676953

**Published:** 2023-06-20

**Authors:** Zulhisyam Abdul Kari, Guillermo Téllez-Isaías, Noor Khalidah Abdul Hamid, Nor Dini Rusli, Khairiyah Mat, Suniza Anis Mohamad Sukri, Muhammad Anamul Kabir, Ahmad Razali Ishak, Nazri Che Dom, Abdel-Wahab A. Abdel-Warith, Elsayed M. Younis, Martina Irwan Khoo, Faizuan Abdullah, Md Shahjahan, Md Fazle Rohani, Simon J. Davies, Lee Seong Wei

**Affiliations:** ^1^Department of Agricultural Sciences, Faculty of Agro-Based Industry, Universiti Malaysia Kelantan, Jeli Campus, 17600 Jeli, Kelantan, Malaysia; ^2^Advanced Livestock and Aquaculture Research Group, Faculty of Agro-Based Industry, Universiti Malaysia Kelantan, Jeli Campus, 17600 Jeli, Kelantan, Malaysia; ^3^Department of Poultry Science, University of Arkansas, Fayetteville, AR 72701, USA; ^4^School of Biological Sciences, Universiti Sains Malaysia, Minden, Pulau Pinang 11800, Malaysia; ^5^Department of Aquaculture, Sylhet Agricultural University, Sylhet 3100, Bangladesh; ^6^Centre for Environmental Health & Safety Studies, Faculty of Health Sciences, Universiti Teknologi Mara, Puncak Alam Campus, Malaysia; ^7^Department of Zoology, College of Science, King Saud University, Riyadh 11451, Saudi Arabia; ^8^Department of Chemical Pathology, School of Medical Sciences, Universiti Sains Malaysia, Kubang Kerian, 16150 Kota Bharu, Kelantan, Malaysia; ^9^Department of Chemistry, Faculty of Science, Universiti Teknologi Malaysia (UTM), 81310 Johor Bahru, Johor, Malaysia; ^10^Laboratory of Fish Ecophysiology, Department of Fisheries Management, Bangladesh Agricultural University, Mymensingh 2202, Bangladesh; ^11^Department of Aquaculture, Bangladesh Agricultural University, Mymensingh 2202, Bangladesh; ^12^Aquaculture and Nutrition Research Unit (ANRU), Carna Research Station, School of Natural Sciences and Ryan Institute, University of Galway, Carna, Co. Galway, Ireland H91 V8Y1

## Abstract

Insects such as black soldier fly larvae (BSFL) are gaining interest among researchers and the aquafeed industry due to the fluctuating price and supply of fish meal (FM). This study evaluated the growth performance, feed stability, blood biochemistry, and liver and gut morphology of Betta splendens using BSFL as an alternative to FM. Five formulated diets were prepared: 0% BSFL, 6.5% BSFL, 13% BSFL, 19.5% BSFL, and 24.5% BSFL. The expansion rate, pellet durability index, floatability, bulk density, and water stability of the prepared feed have been assessed. Except for the diameter of the feed, all the parameters studied differed significantly (*p* < 0.05) across the experimental diets. After 60 days, the fish fed with 13% BSFL had the highest final length, final weight, net weight gain, specific growth rate, weight gain, and gastrointestinal weight, with mean and standard deviation values of 3.97 ± 0.43 cm, 3.95 ± 0.1 g, 2.78 ± 0.1 g, 4.63 ± 0.17, 4.65 ± 0.13, 237.26 ± 7.9%, and 0.04 ± 0.01 mg, respectively. Similar blood haematology and biochemical properties, including corpuscular volume, lymphocytes, white blood cells, red blood cells, haematocrit, albumin, and alkaline phosphatase, were the highest (*p* < 0.05) in the 13% BSFL diet group compared to the other treatment groups. In addition, BSFL had a significant impact (*p* < 0.05) on villus length, width, and crypt depth for the anterior and posterior guts of *B. splendens*. The 13% BSFL diet group had an intact epithelial barrier in the goblet cell arrangement and a well-organized villus structure and tunica muscularis, compared to the other treatment groups. Furthermore, the liver cell was altered with different BSFL inclusions; the 13% FM group demonstrated better nuclei and cytoplasm structure than the other treatment groups. In conclusion, replacing 13% FM with BSFL could improve the growth performance, blood parameters, and liver and intestine morphology of *B. splendens*, thus providing a promising alternative diet for ornamental freshwater fish.

## 1. Introduction

The freshwater ornamental fish represents around 90% in trading activity of the global market [1]. *Betta splendens*, or the Siamese fighting fish, is one of the most popular species among pet lovers because of its colorful appearance and low maintenance [2, 3]. *B. splendens* can be found in several countries, such as Malaysia, China, Singapore, Indonesia, Thailand, Japan, the United States of America (USA), and Mexico. Most importantly, this freshwater ornamental fish species offers a means for income generation even at a small-scale production, thus contributing to the local economy in various countries [4].

In recent years, fish meal (FM) production continues to decrease, urging researchers to find alternatives from other sources such as animal and plants [5–8]. It was estimated that the FM production will not be able to fulfil the global demand by 2050 and the rising FM prices heavily impacted the fish feed production industry [9]. In addition, the partial or complete replacement of FM with other protein sources has been accelerated by the rise in FM cost, reduced availability, irregular supply, and poor quality [10]. Among them, the insect is a promising animal-based protein for FM replacement because of its sustainable growth, environmentally friendly, low operation cost, and highly nutritive [11–15]. *Hermetia illucens* or black soldier fly larvae (BSFL) have been widely studied as a fish meal replacer in many studies and acknowledged as an aquafeed due to the comparable protein content to FM [16]. Despite numerous potential advantages, the use of insect meals may have a disadvantage. Insect meals contain more chitin, which interferes with digestion and nutrient absorption [17]. In addition, whole BSF larva meal has been linked to hepatic steatosis in some studies [18, 19]. Therefore, defatted BSFL has been proposed to replace a whole meal inclusion in the fish feed.

*B. splendens* were chosen in this study because (i) this ornamental fish has become a source of income and a highly preferred hobby for rural people around the world [20] and (ii) ASEAN countries, including Malaysia, are the biggest exporter of ornamental fish like *B. splendens* due to its high profit, short reproductive cycle, and low maintenance [21, 22]. Nutrition requirements within the fish husbandry are crucial for *B. splendens* farming to maintain good growth and health performance [23]. Farmers of *B. splendens* often rely on live foods such as phytoplankton and mosquito larvae [24, 25]. However, these live foods may not be sufficient to meet the nutritional needs of this aquaculture species [26, 27] and involve higher labour costs than the formulated feed [24]. A good diet for ornamental fish should be nutritionally balanced with high palatability and water stability [28, 29]. According to Sipaúba-Tavares et al. [24], *B. splendens* fed with a mixed diet can improve the survival rate and also improve the culture management for fish production. At the moment, there are very limited studies on the minimum nutrient requirement of *B. splendens* [30]. However, the protein requirement for the ornamental fish range is 30–48.6% depending on the species [31, 32]. Furthermore, a high protein requirement of the fish means that more fish meal has to be added. This leads to unsustainable use of natural resources, including for the ornamental fish industry, especially tropical species like Siamese fighting fish, which may not require high-quality fatty acids and amino acids from the fish meal. In recent years, the use of BSFL meal in aquafeed has been extensively studied in many farmed fish such as salmon [33], rainbow trout [34], grouper [35], tilapia [36], mirror carp [37], and African catfish [38]. A similar trend was observed in ornamental fishes, including lemon fin barb [39], goldfish [40], zebrafish [41, 42], and discus fish [43].

Thus, the current study assessed the potential of BSFL as an FM substitute at various percentages on Siamese fighting fish's growth performance, feed stability, haematology, blood biochemistry, and liver and stomach morphology in an aquarium environment.

## 2. Materials and Methods

### 2.1. Animal Ethics Approval

This study has been approved by the Animal Ethics Committee of Faculty of Agro-Based Industry, Universiti Malaysia Kelantan (UMK/FIAT/ACUE/UG/034/2021). All experiment protocol was conducted per the committee's comments and approval.

### 2.2. Preparation of BSFL Powder

**Figure 1:**
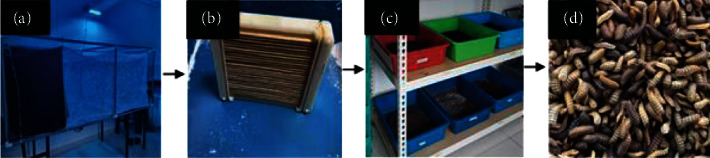
The BSF production under laboratory conditions: (a) BSF cage, (b) BSF egg collection, (c) composting container, and (d) harvested BSFL.

The black soldier fly (BSF) colony was established and maintained at room temperature throughout the study, located at the UiTM Insectarium, Faculty of Health Sciences, Universiti Teknologi MARA (UiTM), Malaysia. The BSF was raised in a cage (6 × 3 × 3 ft) with a multispectrum light to simulate sunlight and promote the mating process. Two days later, the BSF eggs were retrieved and placed in a rectangular plastic tray to hatch, producing BSF neonates (see Figure 1). Subsequently, the BSF neonates were transferred to the feeding container in a naturally aired, shaded location after five days to start the composting process. After two weeks, the growing larvae using food waste and the waste mixture were collected and separated. The larvae were dried and processed into powder at a maximum temperature of 80°C before being incorporated into the experimental diets and tested for proximate analysis.

### 2.3. Preparation of Experimental Diets

The BSFL powder was incorporated into experimental diets at varying levels as the following: T1 (0% BSFL), T2 (6.5% BSFL), T3 (13% BSFL), T4 (19.5% BSFL), and T5 (24.5% BSFL) as a FM replacement. The T1 group acted as the control group with 0% BSFL. The BSFL powder was added to other ingredients, including FM, soybean meal, wheat flour, vitamin, mineral premix, rice bran, and molasses (binder) in an HDPE container according to the formulated experimental diets. The mixture was later pelleted through the extruder to produce 1.5 to 2 mm diameter suitable for *B. splendens* mouth size. The pellets were then sundried until 10% moisture content and stored safely at -20°C until use. The composition and proximate analysis of the formulated feed (Table 1) and BSFL (Table 2) were determined as recommended by [44].

### 2.4. Experimental Diet Physiological Characteristics

The expansion ratio, bulk density, pellet durability index, water stability, and floatability of the experimental diets were determined before the animal feeding trial according to the method by Zulhisyam et al. [29], Khater et al. [45], and Rawski et al. [46] as follows:

- Pellet durability index (%) = (weight of pellet remains on the sieve/total weight of pellets) × 100
- Expansion rate (%) = [pellet diameter (mm)/matrix diameter (mm)] × 100
- Floatability (%): the total number of floating pellets in the water was recorded. The mean of the floating pellet was calculated and expressed as a percentage of the total pellets at the beginning of the experiment
- Bulk density (kg/dm^3^) = sample weight/dm^3^
- Organoleptic/palatability test: feed color and odor were assessed via visual observation
- Water stability (%) = (final sample weight − initial sample weight)/(initial sample weight) × 100

### 2.5. Animal Study

A total of 60 of two-month-old Siamese fighting fish with an average weight of 1.1 g were purchased from a pet shop in Bachok, Kelantan (Sentosa Pet Shop Enterprise). The fishes were fed with floating commercial ornamental fish feed (2 mm diameter) formulated as 32% crude protein, 4% fat, and 11% moisture (brand: Dindings, Malaysia). The acclimatization was performed for 10 days before dividing the fish into 30 indoor tanks (60 cm × 30 cm × 30 cm). Two fishes were housed in each tank equipped with continuous air supply and using chlorine free water, as recommended by Srikrishnan et al. [47], while six replicates were in each treatment group. The experiment was conducted for 60 days, where the fish were given the respective feed to satiety twice daily (average of 126.6 g for 60 days/treatment). In addition, the water parameters in the tank were maintained under the following conditions: pH (7.2 ± 0.9), temperature (24.2 ± 1.1°C), dissolved oxygen (5.8 ± 1.2 mg/L), total ammonia (1.13 ± 0.2 mg/L), nitrite (0.14 ± 0.21 mg/L), alkalinity (60.0 ± 12.4 mg CaCO_3_/L), and hardness (94.8 ± 1.9 mg CaCO_3_/L).

### 2.6. Growth Performance of B. splendens

The growth performance of *B. splendens* was recorded following Thongprajukaew et al. [48] and Kari et al. [49]. First, the weight was measured using an electronic balance. The net weight gain (g), specific growth rate, weight gain (g), feed conversion rate, and survival rate (%) were calculated using the specific formula.

- Net weight gain (g) = final body weight − initial body weight
- Specific growth rate (%) = [(ln final weight–ln initial weight)/experiment duration] × 100
- Weight gain (%) = (final weight − initial weight)/initial weight) × 100
- Feed conversion rate = total feed consumption/weight gain
- Survival rate (%) = (total surviving fish/total number of fishes at the beginning of the feeding trial) × 100

### 2.7. Histological Examination of B. splendens Gut and Liver

Three replicates from each group were collected randomly and dissected to obtain their gut and liver samples. The tissues were immediately fixed in 10% neutral-buffered formalin solution individually. Subsequently, 1 mm transverse sections from each gut and liver segment were cut and subjected to tissue processing using a series of graded ethanol for dehydration, cleared in xylene, and embedded in paraffin blocks. The blocks were then sectioned transversely (5–8 *μ*m) and mounted on glass slides before oven-drying overnight at 40°C. The sections were later stained with haematoxylin and eosin and evaluated under a light microscope (Olympus BX43, Japan). The images were captured and digitized using an image capture analysis system (Cellsens software, Netherlands) to assess the fish's villus length, width, and crypt depth.

### 2.8. Haematological Parameters

Three replicates from each group were collected randomly, placed in plastic tanks, and deprived of food for 5 h. The fishes were anesthetized using tricaine methanesulfonate (MS-222), 1 ml for 1 litre water; a 1 cc sterile syringe was used to collect blood from the midventral line behind the anal fin and placed in heparinized tubes (green and yellow caps) as described by Zang et al. [50]. The blood samples (150 *μ*l) were subjected to a complete blood count test to determine the blood haematological parameters using the automatic haematology analyzer (Mythic 18 Vet, USA). Meanwhile, a 150 *μ*l blood serum sample was pipetted onto cassettes containing reagents of the individual biochemistry blood tests (brand: IDEXX, USA) and automatically detected by the VetTest analyzer (brand: IDEXX, USA) except for globulin. The globulin was determined by subtracting the albumin from the total protein levels.

### 2.9. Statistical Analysis

All data collected were analysed using the Statistical Package for the Social Sciences (SPSS) version 20.1 (IBM, USA). All results were subjected to a normality test before further analysis. The results that passed the normality test were further analysed with the parametric test, where the homogeneity of variance was tested with Levene's test. Then, a one-way analysis of variance (ANOVA) was used to compare all study parameters between all treatment groups. For post hoc analysis, data were analysed using Duncan's test if homogeneity of variance could be met. If the variance was heterogeneous, Tamhane's T2 test was used for post hoc analysis. The results were reported as mean ± SD and a significance level of *p* < 0.05. Data that did not conform to the normal distribution were subjected to a nonparametric test using the Kruskal-Wallis test and two independent tests to detect significant differences between treatments and indicated in the heading of the presented results.

## 3. Results

### 3.1. Physical Characteristics of Experimental Diets

The expansion rate, pellet durability index, floatability, bulk density, and water stability of the experimental diets were significantly different (*p* < 0.05). There was an increasing trend in expansion rate, bulk density, and water stability for all treatments throughout the feeding trial. No significant difference was detected for all treatments of feed diameter with a value of 1.2 ± 0.0 mm (Table 3).

The feed color, odor, and palatability of the experimental diets are detailed in Table 4. The findings indicated that feed color and odor varied between treatments. As the percentage of BSFL increased in the diets, the feed color appeared yellowish with a more robust flavor. Meanwhile, there was a similar trend in terms of palatability for all experimental diets. Groups T1, T2, T3, and T4 consumed the whole given feed within less than 5 minutes, whereas group T5 diet group fed on less than 75% of the pellet in 5 minutes.

### 3.2. Growth Performance of B. splendens

There were significant differences (*p* < 0.05) in the growth performance parameters of final weight (g), final length (cm), net weight gain (g), weight gain (%), specific growth rate (%), and gastrointestinal weight (mg) as shown in Table 5. The highest final length, final weight, net weight gain, specific growth rate, and gastrointestinal weight were recorded in the T3 diet group at 3.967 ± 0.176 cm, 3.952 ± 0.042 g, 2.78 ± 0.04 g, 4.64 ± 0.06, and 0.039 ± 0.001 mg, respectively. Conversely, T3 had the lowest food conversion rate (FCR) at 0.45 ± 0.06. Nevertheless, there were no significant differences (*p* > 0.05) in survival rate between treatment groups, with T3 charting the highest value at 100 ± 0.00%.

### 3.3. Haematological Parameters of B. splendens

Table 6 shows the haematological indices of *B. splendens* fed with BSFL at varying levels. There were significant differences (*p* < 0.05) in haematological parameters between all treatment groups. Fishes in the T3 group recorded the highest white blood cell (WBC), lymphocytes (LYM), red blood cell (RBC), haematocrit (HCT), and mean corpuscular volume (MCV) at 18.4 ± 0.4 10^3^/*μ*l, 61.7 ± 0.0%, 6.8 ± 0.2 10^6^/*μ*l, 45 ± 0.57%, and 79.7 ± 1.1 *μ*m^3^, respectively. Meanwhile, significant variations (*p* < 0.05) were observed for blood granulocytosis (GRA), red cell distribution width (RDW), platelet (PLT), and platelet distribution width (PDW) levels among the treatments, but no specific trend was identified in this study. Furthermore, the T5 group had the highest values for GRA (66 ± 0.7%), RDW (14.6 ± 0.4%), PLT (62.2 ± 0 *μ*l), and PDW (8.7 ± 0.2%).

### 3.4. Blood Biochemical Parameters of B. splendens

Table 7 demonstrates the blood biochemistry for all treatment groups at the end of the feeding trial. Alkaline phosphatase (ALKP), alanine aminotransferase (ALT), blood urea nitrogen (BUN), and total protein (TP) were significantly lower (*p* < 0.05) in the T1 diet group at 6.25 ± 0.05 *μ*/l, 21.23 ± 0.32 *μ*/l, 1.17 ± 0.05 mg/dl, and 1.13 ± 0.06 g/dl, respectively. Meanwhile, the T3 group recorded the highest ALB and ALKP levels at 1.24 ± 0.06 g/dl and 7.67 ± 0.32 *μ*/l, respectively. In addition, the blood urea nitrogen (BUN), total protein (TP), calcium ion (Ca^2+^), cholesterol (CHOL), and creatine (CREA) were significantly different between groups without any particular trend.

### 3.5. Midintestine and Liver Morphology of B. splendens

**Figure 2:**
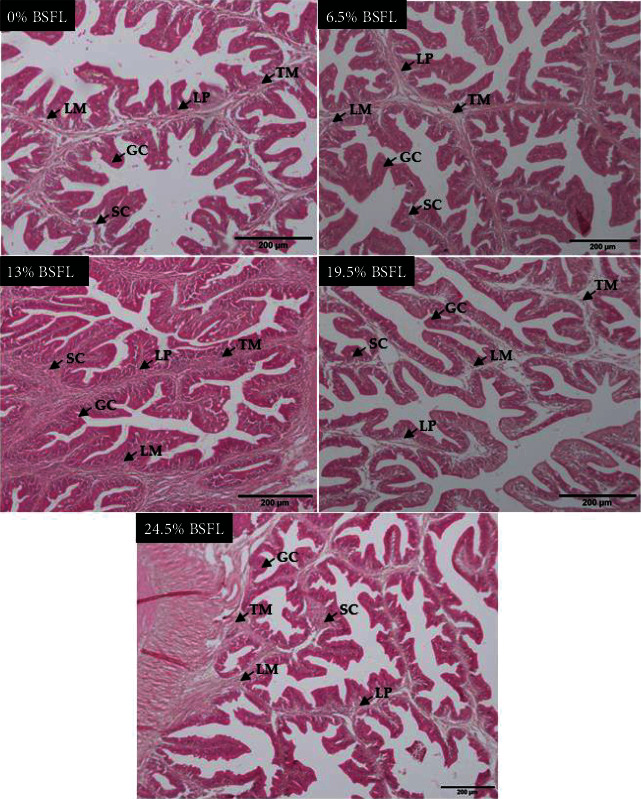
Morphology of the *B. splendens* distal intestines under the light microscope after the feeding trial. All images were of 40x magnification, with a scale bar of 200 *μ*m. Histological alterations were evident in lamina propria (LP), lamina epithelial mucosae (LM), stratum compactum (SC), goblet cells (GC), and tunica muscularis (TM).

Generally, histological alterations were identified in the intestines of *B. splendens* fed with BSFL at varying levels. Structural changes were evident in the fish gut in the lamina propria, lamina epithelial mucosae, tunica muscularis, stratum compactum, villus, and goblet cells. Furthermore, the T3 diet group exhibited intact epithelial barrier with goblet cell arrangement and well-organized villus and tunica muscularis structures than the other treatment groups. Meanwhile, moderate histological changes were detected in the T2 and T4 groups than in T5, but all the dishes in the respective groups had regularly distributed lamina epithelial mucosae, tunica muscularis, and large goblet cells (see Figure 2).

**Figure 3:**
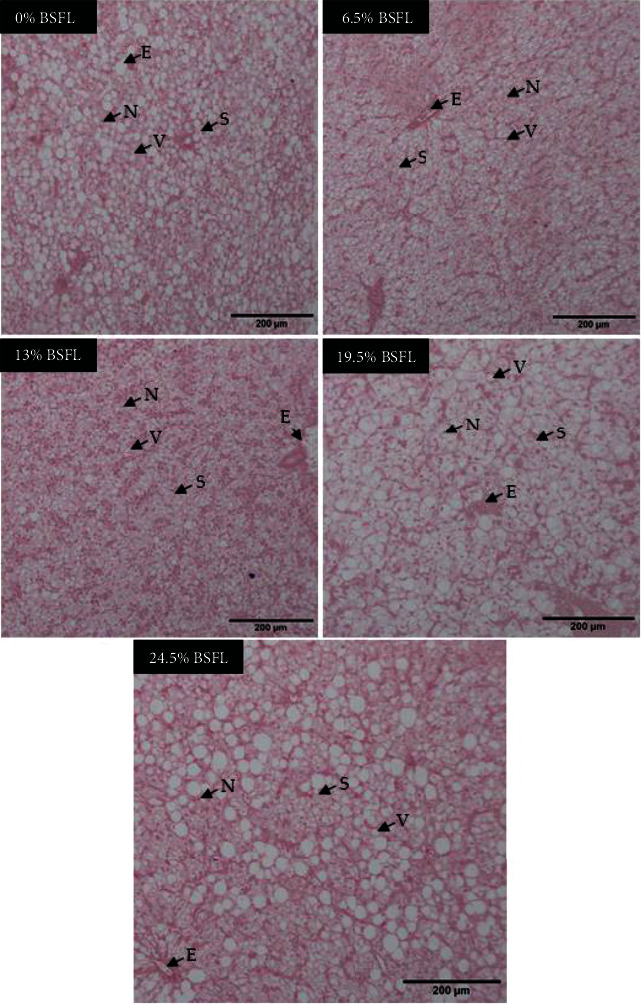
Morphological appearance of *B. splendens* liver after the feeding trial viewed under the light microscope. All images were of 40x magnification, with a scale bar of 200 *μ*m. Histological alterations were evident in sinusoid (S), vacuole (V), nucleus (N), and erythrocytes (E).

The fish's liver cells also underwent changes in sinusoid, nucleus, and vacuole when fed with BSFL at varying levels (see Figure 3). For instance, the T3 diet group illustrated improved nuclei and cytoplasmic structure than the other treatment groups. In contrast, there were marked nuclei, cytoplasm atrophy, and unorganized hepatic cell cords in the T4 and T5 diet groups. Moreover, an increase in BSFL incorporation in the diets generally resulted in more vacuolar cytoplasms, except for the T3 group, which did not display these abnormalities.

### 3.6. Villus Length, Width, and Crypt Depth Estimation of B. splendens

The histomorphology of the anterior and posterior gut of *B. splendens* was analysed under the light microscope (see Table 8). It was found that BSFL protein supplementation at varying levels significantly affected (*p* < 0.05) the villus length, width, and crypt depth in the fish's anterior and posterior gut.

## 4. Discussion

Currently, the aquafeed technology advancement is moving in tandem with the aquaculture industry growth, utilizing extrusion procedures to improve the digestibility and quality of the fish pellets [51]. Several physical characteristics and stability are crucial in feed formulation to ensure optimal packaging, transport, and pneumatic feed delivery systems [45, 52]. This present investigation showed that the physical properties of feed pellets significantly varied among the various test groups of BSFL. These outcomes may be due to the identical pellet quality and the percentage of BSFL inserted. According to Singh et al. [53], bulk density refers to the extent of puffing of the extruded material that influences the packaging material, design, and feed storage [54]. The recommended bulk density of fish feed ranges from 267.11 to 711.35 kg m^−3^ [45]. The current study findings indicated that the bulk density of the fish pellets increases with the expansion rate, which coincided with earlier research [45, 55, 56]. Furthermore, an earlier study discovered an association between floatability and bulk density [57], where the former significantly decreased as the BSFL composition increased in the fish pellet. Floatability is also affected by the source of materials, such as carbohydrates and binding agents [58].

A good physical structure is vital in ensuring the maximum floatability of fish pellets. Several factors affect the PDI, water stability, and floatability of fish pellets, such as binder material [58], pellet formulation and size [56], and the feed manufacturing method [59, 60]. The present findings exhibited that floatability decreases with increasing levels of BSFL incorporated in the fish feed. Furthermore, Irungu et al. [61] concluded that floatability increased with higher moisture content, contradicting the results of this study where floatability was reduced with the increase in moisture content. Meanwhile, Obaldo et al. [62] reported pellets with a larger diameter demonstrated a higher degree of water stability. Pellet size and texture should be suitable for the fish's mouth gap to maximize feed consumption and minimize wastage [63]. It was reported that the ideal feed diameter for *B. splendens* is 1.2 mm [64].

The feed color and odor for all experimental diets differed from one another. Mainly, fish feed that is brown and deep brown might indicate microbial contamination [65]. In the present study, the experimental diets had strong odors due to BSFL and molasses content, contrary to T1 (control diet). Furthermore, water color changes were prominent with increasing levels of BSFL in the feed formulation, from yellowish to darker yellowish brown. This finding is aligned with Shamsuddin et al. [56], who reported that water color changes from yellowish green to turbid yellow when shrimps were fed with palm kernel-based feeds. In addition, palatability is crucial in maintaining feed quality and improving feeding for aquaculture species. Several factors influence feed palatability, including feeding behaviour, nutritional and chemical content, fish's nutritional requirement, and molecular weight of organic compounds such as nucleotides and organic acids [65, 66]. Naturally, the fish utilize their senses, such as smell, taste, and sight, in feed selection and consumption. Nevertheless, it is essential to conduct more studies to standardize the analytical tools in assessing feed quality and to have a deeper understanding of the association between the physical and nutritional characteristics of the feed.

Siamese fighting fish responded positively to lower levels of fish meal replacement with BSFL meal on growth parameters such as SGR and weight gain, with 13% BSFL meal performing best. When increased to 19.5% and higher, BSFL began to have a detrimental impact on Siamese fighting fish's growth performance. This observation is in line with most of the growth performance reported in fish species such as Atlantic salmon [67], barramundi [68, 69], Nile tilapia [26, 70], rainbow trout [71], Siberian sturgeon [72], turbot [17], yellow catfish [73], and zebrafish [19].

As for FCR, the lowest value was calculated for the diet containing 13% BSFL meal, consistent with Siamese fighting fish's growth performance. The feed conversion ratio is one of the vital parameters in evaluating the potential use of BSFL meal as a substitute for fish meal in fish feed production, and it seems to be a quantity-dependent response with the BSFL inclusion. In this study, we observed that the FCR value decreased with an increase of BSFL up to 13% in dietary inclusion. A further increase in the proportion of BSFL leads to a rise in the FCR value. This pattern is similar to the study using African catfish by Fawole et al. [74], where they reported that FCR value continues to decrease with an increase in the proportion of BSFL meal up to a value of about 10%. The efficiency of the diet decreased once the BSFL meal content was increased to 17%. A different study in African catfish by Mohd Azri et al. [38] showed that the FCR value of feed containing 20% BSFL meal was similar to that of the control but not at 30% dietary inclusion, where the FCR value was negatively affected. In tilapia, 10% BSFL meal inclusion resulted in an FCR comparable to the control group [26, 75]. Several factors contribute to poor growth performance and FCR in the fish fed higher levels of BSFL meal, one of which could be chitin. According to Priyadarshana and Ruwandeepika [76], poor growth performance is usually attributed to poor digestibility due to chitin in BSFL. Chitin is an exoskeleton for insects and a cellulose-like biopolymer. Chitinase is an enzyme that breaks down the chitin structure in the digestive tract. Insect meal generally contains chitin, which has been shown to be indigestible to many fish species [77]. Although chitinase has been found in fish, it is unlikely that the amount produced is sufficient to digest the chitin making the ability to digest chitin vary from fish species to fish species [78, 79].

Overall, the dietary inclusion of BSFL meal in feed for Siamese fighting fish did not have an adverse effect on their survival. Several studies have reported similar rates of survival in their studies. For example, Dumas et al. [80] reported survival of rainbow trout above 95% in rainbow trout using up to 25% of BSFL meal in the formulation. In a different study, Kuo et al. [81] tested BSFL meal in the Japanese eel diet and reported a survival rate above 95%. Although in Betta, at 24.5%, BSFL meal inclusion showed an almost 20% mortality rate, it could be due to the feed's palatability, but further studies are needed to confirm this.

Haematological indices are principal in aquaculture nutrition and are essential in evaluating formulated feed efficacy, physiological stress, and fish health and growth performance [82, 83]. In the present study, the significant differences in WBC and RBC levels indicated that varying physiological properties could increase antigen distribution [84] and improve oxygen level and anti-infection ability, thus improving fish growth and health performance. The T3 group recorded the highest WBC and RBC values in this study. On the contrary, Tippayadara et al. [85] reported that BSFL as an FM replacement did not influence an aquaculture species WBC and RBC levels. In addition, haematocrit (HCT) is a valuable factor in assessing fish feed health and antinutritional toxicity [86]. The current study demonstrated no specific trend in HCT value for fish fed with 13% BSFL diet. The inconsistency in HCT values suggested the ability of BSFL to regulate protein involved in fish growth and health status [87]. Moreover, differences in haemoglobin (HGB), mean corpuscular haemoglobin concentration (MCHC), and mean corpuscular haemoglobin (MCH) values between the treatment groups are reflective of the good fishes' health status. Conversely, no specific trend was identified for the fish's ALB levels, but the 13% BSFL group recorded the highest ALB level. Based on the study by Nya and Austin [88], ALB and GLOB contributed to a healthy system and function of plasma carriers. In addition, ALB and ALT are indicators of hepatocyte injury and liver necrosis [89]. The present findings showed that the 24.5% BSFL recorded the highest ALT compared to the other treatment groups. Furthermore, changes in ALT enzyme activities in the blood are an indication of stress, such as tissue impairment in an aquaculture species [87].

Intestinal and hepatic morphology is an essential aspect in assessing the alterations made by nutrients digested and energy stored by fish because the tissue structure of these organs is responsive to dietary input. In this study, the 13% BSFL-fed fish had an intact epithelial barrier with a highly organized villus structure, goblet cell arrangement, and tunica muscularis compared to the other treatment groups. The precise alteration of villus length and width by BSFL meal is inconclusive. Several studies mentioned mixed responses. Most of the studies reported that the inclusion of BSFL meal altered neither the physical size of the villi nor the growth performance of the species studied, such as clown fish [90], gilt-head sea bream [91], rainbow trout [80], barramundi [92], Siberian sturgeon [93], and olive flounder [94]. In a different study, 19% of BSFL meal inclusion did not alter the length of the villi in barramundi, but the presence of lupin kernel meal did [68]. In Japanese eels, villus length was significantly shortened when the inclusion level of BSFL in the feed formulation was at 45% [81]. This response contrasts with Siamese fighting fish's finding in the present study in which the 13% and 19.5% BSFL inclusion caused villus elongation and anterior/posterior width extension. Rawski et al. [95] stated that gut histology changes to maintain nutrient digestibility or increase micro- and macronutrient absorption, which is expected in the intestine due to observing improvements in growth performance. We discovered that more substantial substitutions of fish meal for BSFL meal had a detrimental effect on villus structure, causing the villi's width to decrease. Cummins et al. [77] reported similar findings as the current study, where the rise in BSFL level in fish feed contributed to the deterioration in the histological structure of the intestine in white shrimp. The extension of villus length and width are also indicators of gut health in which imbalanced dietary nutrition may result in the degeneration and erosion of the villus structure [19, 72, 96].

Goblet cells (GCs) are single-cell glands that produce and secrete mucin. Mucin forms a mucus layer, which can separate the materials in cavities from the intestinal epithelium and prevent the invasion of pathogenic microorganisms in various ways. In this study, we found that the density of GCs in the villi increases with an increasing level of BSFL meal inclusion of up to 13% of BSFL meal. Further inclusion caused a reduction in the density of GCs in the villi. A similar observation was also reported in Japanese eel [81], where the density of GC distribution reduced at 27.5% of dietary inclusion. However, that was not the case in olive flounder, where the GC density was not affected by BSFL meal inclusion [94].

The liver is an energy storage in which the metabolism of nutrients such as carbohydrates, lipids, and proteins will involve liver function. In fish nutrition, hepatic steatosis is one of the indicators of farmed fish health, primarily when alternative ingredients have been used in the formulation [96–98]. BSFL meal inclusion has often been associated with hepatic steatosis in fish [99]. Hence, the use of defatted BSFL meal has been suggested [100]. In general, no hepatic steatosis and vacuolisation due to fat accumulation were observed in the Siamese fighting fish fed with control and BSFL meal diet except for the diet with a 19.5% inclusion level. Fish provided with 13% BSFL meal exhibited improved nuclei and cytoplasm arrangement compared to the other treatment groups. At 19.5% and 24.5% BSFL inclusion levels, the cytoplasm and nuclei were atrophied and unorganized hepatic cell cords and dark-staining nuclei and cytoplasm of hepatocytes (see Figure 3). Fischer et al. [97] found that largemouth bass fed with black soldier fly prepupae (BSFP) caused liver inflammation and necrosis in one-third of the fish samples. However, a study on Atlantic salmon shows that at a low-level inclusion that was 3.5%, BSFL meal did not cause hepatic steatosis [94], but hepatic steatosis did not occur in Siberian sturgeon fed with defatted BSFL [100].

Several limitations were identified in the present study. First, *B. splendens* feeding behaviour changes from the larval to the maturation stage and is dependent on visual ontogeny. In the wild, these species are carnivorous and feed on mosquito larvae. Furthermore, young *B. splendens* that consumed animal- and plant-based protein feed demonstrated improved growth and female ovary development [101]. In the present study, the low final weight of *B. splendens* could be associated with the fish's nature and feed intake. Nonetheless, the current findings act as preliminary data for future research on the nutritional requirement of aquaculture species at the larval stage. In summary, the insights from this study are potentially helpful for future studies and feed producers since *B. splendens* is a commercially important species.

## 5. Conclusions

The rising demand for fish pellets from the global aquaculture sector has highlighted the importance of BSFL as a raw material in feed production. The present study utilized a feeding trial to assess the impacts of BSFL on *B. splendens* growth performance and health status. It was discovered that replacing FM with BSFL resulted in significant improvements in the growth and health status of *B. splendens.* Furthermore, the study findings indicated that BSFL inclusion at 13% in fish feed could enhance the *B. splendens* growth performance, blood haematology, and liver and gut morphology. This research provided insights about the animal-based proteins such as BSFL, contributing to low-cost and healthy fish feed production. In addition, the study findings will aid future researchers in critical areas, such as BSFL delivery techniques of fish feed pellets in ornamental fish. In conclusion, BSFL is a promising animal-based raw material and protein supplement to enhance *B. splendens* growth and health status when incorporated at 13% of the fish diet.

## Figures and Tables

**Table 1:** Composition and proximate analysis (g/100 g dry weight) of the experimental diets for *B. splendens.*

	Experimental feed				
	T1	T2	T3	T4	T5
Ingredients (%)					
Fish meal^1^	31	24.5	18	11.5	6.5
BSFL^2^	0	6.5	13	19.5	24.5
Soybean meal	25	25	25	25	25
Wheat flour	10	10	10	10	10
Vitamin mineral premix^3^	2	2	2	2	2
Molasses	4	4	4	4	4
Rice bran	28	28	28	28	28
Total	100	100	100	100	100

Proximate analysis (%)					
Crude fiber	2.1	2	2.1	2	2.1
Crude fat	3.6	3.2	3.8	3.5	3.7
Crude protein	33.7	33.5	33.3	33.7	33.2
Moisture	6.1	6.3	6.8	6.9	6.8
Ash	6.7	6.4	7.1	7.1	7.4
Carbohydrate	47.8	48.6	46.9	46.8	46.8
Energy value (kcal/100 g)	358.4	357.2	355	353.5	354.5

^1^Danish fish meal. ^2^Black soldier fly larvae. ^3^g/kg premix: vitamin C, KCL, 90; KI, 0.04; CaHPO_4_.2H_2_O, 500; NaCl, 40; CuSO_4_.5H_2_O, 3; ZnSO_4_.7H_2_O, 4; CoO_4_, 0.02; FeSO_4_.7H_2_0, 20; MnSO_4_.H_2_O, 3; CaCo_3_, 215; MgOH, 124; Na_2_SeO_3_, 0.03; NaF, 1. Brand: Bar-Magen. Treatment groups: T1 (0% BSFL), T2 (6.5% BSFL), T3 (13% BSFL), T4 (19.5% BDFL), and T5 (24.5% BSFL).

**Table 2:** Proximate analysis (%) of BSFL used for feed formulation.

Proximate analysis	Value (%)
Crude fiber	8.3
Crude fat	11.3
Crude protein	48.6
Moisture	5.9
Ash	21.6

**Table 3:** Physical properties of the experimental diets (*n* = 10).

Parameters	Experimental diet				
	T1	T2	T3	T4	T5
ER (%)	1.1 ± 0.1^a^	12.8 ± 0.9^b^	22.6 ± 0.4^c^	33.5 ± 1.3^d^	55.2 ± 1.00^e^
PDI (%)	98.3 ± 0.2^e^	92.5 ± 0.6^d^	95.3 ± 0.3^c^	91.3 ± 1.1^b^	90.2 ± 0.3^a^
Floatability (%)	99.2 ± 0.1^d^	98.4 ± 0.3^c^	97.3 ± 0.1^b^	96.9 ± 0.2^b^	95.8 ± 0.5^a^
BD (kg·m^-3)^	470.8 ± 0.6^a^	542.7 ± 5.3^b^	610.1 ± 1.9^c^	623.6 ± 0.4^d^	652.8 ± 4.1^e^
FD (mm)	1.2 ± 0.0	1.2 ± 0.0	1.2 ± 0.0	1.2 ± 0.0	1.2 ± 0.0
WS (%)	62.8 ± 0.5^a^	79.3 ± 0.2^b^	84.6 ± 0.5^c^	84.7 ± 1.00^c^	92.1 ± 0.1^d^

Note: different superscripts in each row indicate a significant difference (*p* < 0.05). Abbreviations: PDI = pellet durability index; ER = expansion rate; BD = bulk density, FD = feed diameter; WS = water stability. Treatment groups: T1 (0% BSFL), T2 (6.5% BSFL), T3 (13% BSFL), T4 (19.5% BDFL), and T5 (24.5% BSFL).

**Table 4:** Organoleptic and palatability characteristics of experimental diets.

Parameters	Experimental feed				
	T1	T2	T3	T4	T5
Feed color	Ash	Turbid yellowish black	Brown yellowish black	Deep yellowish black	Deeper yellowish black
Feed odor	Fishy odor	Characteristic flavor	Powerful flavor	Strong flavor	Stronger flavor
Palatability	++++	++++	++++	++++	+++

Note: + = fish consumed less than 25% of given feed in 5 minutes; ++ = fish consumed less than 50% of given feed in 5 minutes; +++ = fish consumed less than 75% of given feed in 5 minutes; ++++ = fish consumed the whole given feed in 5 minutes. Treatment group: T1 (0% BSFL), T2 (6.5% BSFL), T3 (13% BSFL), T4 (19.5% BDFL), and T5 (24.5% BSFL).

**Table 5:** Growth performance of *B. splendens* after the 60-day feeding trial (*n* = 6).

Parameter	Experimental feed				
	T1	T2	T3	T4	T5
IL (cm)^∗^	1.130 ± 0.028	1.123 ± 0.029	1.033 ± 0.021	1.083 ± 0.030	1.094 ± 0.051
IW (g)^∗^	1.165 ± 0.002	1.168 ± 0.002	1.168 ± 0.003	1.165 ± 0.002	1.167 ± 0.002
FL (cm)^#^	3.087 ± 0.061^a^	3.233 ± 0.061^bc^	3.967 ± 0.176^c^	2.367 ± 0.041^b^	3.067 ± 0.105^a^
FW (g)^∗^	2.885 ± 0.220^a^	2.975 ± 0.234^ab^	3.952 ± 0.042^b^	2.712 ± 0.036^a^	2.307 ± 0.011^a^
NWG (g)^∗^	1.72 ± 0.22^b^	1.81 ± 0.23^b^	2.78 ± 0.04^c^	1.70 ± 0.16^b^	1.14 ± 0.01^a^
SGR^#^	2.87 ± 0.36^b^	3.01 ± 0.39^bc^	4.64 ± 0.06^c^	2.58 ± 0.06^b^	1.90 ± 0.02^a^
TF (g)^#^	117.33 ± 5.85^b^	110.17 ± 2.75^b^	112.67 ± 4.66^b^	235.83 ± 21.38^c^	57.00 ± 4.55^a^
FCR^#^	0.85 ± 0.09^b^	0.75 ± 0.22^ab^	0.45 ± 0.10^a^	1.67 ± 0.05^b^	0.58 ± 0.02^ab^
GW (g)^∗^	0.023 ± 0.002^b^	0.024 ± 0.001^b^	0.039 ± 0.001^c^	0.021 ± 0.001^b^	0.013 ± 0.001^a^
DI^#^	1.52 ± 0.30	1.45 ± 0.23	1.40 ± 0.06	1.22 ± 0.12	1.15 ± 0.06
SR (%)	91.7 ± 8.3	91.7 ± 8.3	100.0 ± 0.0	91.7 ± 8.3	83.3 ± 10.5

Note: different superscripts in each row indicate a significant difference (*p* < 0.05). Asterisk (^∗^) indicates that nonparametric analysis was performed. The octothorpe (#) indicates that a parametric test was applied. Abbreviations: IL = initial length; FL = final length; IW = initial weight; FW = final weight; NWG = net weight gain; SGR = specific growth rate; FCR = feed conversion rate; GW = gastrointestinal weight; SR = survival rate; TF= total feed; DI= digestosomatic index. Treatment group: T1 (0% BSFL), T2 (6.5% BSFL), T3 (13% BSFL), T4 (19.5% BDFL), and T5 (24.5% BSFL).

**Table 6:** Blood parameters of *B. splendens* after the 60-day feeding trial (*n* = 3).

Item	Diets (g/kg)				
	T1	T2	T3	T4	T5
WBC (10^3^/*μ*l)	8.3 ± 0.01^b^	11.5 ± 0.4^c^	18.4 ± 0.4^e^	14.9 ± 0.8^d^	7.3 ± 0.2^a^
LYM (%)	21.5 ± 0.3^b^	42.5 ± 0.4^c^	61.7 ± 0.3^e^	58.3 ± 0.1^d^	19.7 ± 0.5^a^
MON (%)	4.9 ± 0.6^a^	4.5 ± 0.1^a^	8.8 ± 0.15^c^	9.8 ± 0.49^d^	5.4 ± 0.2^b^
GRA (10^3^/*μ*l)	49.3 ± 1.2^b^	51.4 ± 0.9^c^	43.7 ± 0.4^a^	55 ± 0.2^d^	66 ± 0.7^e^
RBC (10^6^/*μ*l)	4.2 ± 0.6^a^	5.3 ± 0.2^b^	6.8 ± 0.2^d^	5.8 ± 0.2^c^	4.2 ± 0.2^a^
HGB (g/dl)	8.6 ± 0.2^b^	8.2 ± 0.1^b^	9.5 ± 0.1^c^	9.6 ± 0.3^c^	7.3 ± 0.5^a^
HCT (%)	24.1 ± 0.2^a^	40.5 ± 0.5^c^	45 ± 0.57^d^	29.0 ±0.7^b^	40.87 ± 0.71^c^
MCV (*μ*m^3^)	29.9 ± 0.2^a^	42.4 ± 1.5^b^	79.7 ± 1.1^d^	58.2 ± 2.7^c^	30.1 ± 0.5^a^
MCH (pg)	23.9 ± 0.5^b^	29.8 ± 1.0^c^	37.9 ± 1.0^d^	39.4 ± 0.7^e^	20 ± 0.1^a^
MCHC (g/dl)	28.1 ± 0.2^b^	26.8 ± 0.2^a^	31.8 ± 0.5^c^	32.5 ± 0.4^c^	26.7 ± 0.6^a^
RDW (%)	12.3 ± 0.3^a^	13.3 ± 0.4^b^	12.3 ± 0.2^a^	13.8 ± 0.2^b^	14.6 ± 0.4^c^
PLT (10^3^/*μ*l)	59.9 ± 0.3^c^	54.9 ± 0.4^b^	49.7 ± 1.1^a^	61.3 ± 0.7^d^	62.2 ± 0.1^d^
MPV (*μ*m^3)^	6.4 ± 0.1^a^	7.0 ± 0.1^b^	8.4 ± 0.3^c^	8.7 ± 0.1^d^	6.3 ± 0.1^a^
PCT (%)	0.1 ± 0.0^c^	0.1 ± 0.0^d^	0.1 ± 0.0^b^	0.1± 0.0^e^	0.04 ± 0.1^a^
PDW (%)	6.9 ± 0.1^b^	6.1 ± 0.1^a^	6.1 ± 0.2^a^	6.7 ± 0.1^b^	8.7 ± 0.2^c^

Note: different superscripts in each row indicate significant differences (*p* < 0.05). Abbreviations: WBC = white blood cell; LYM = lymphocytes; MON = monocytes; GRA = granulocytosis; RBC = red blood cell; HGB = haemoglobin; HCT = haematocrit; MCV = mean corpuscular volume; MCH = mean corpuscular haemoglobin; MCHC = mean corpuscular haemoglobin concentration; RDW = red cell distribution width; PLT = platelet; MPV = mean platelet volume; PCT = procalcitonin; PDW = platelet distribution width. Treatment group: T1 (0% BSFL), T2 (6.5% BSFL), T3 (13% BSFL), T4 (19.5% BDFL), and T5 (24.5% BSFL).

**Table 7:** Blood biochemistry results of *B. splendens* after the 60-day feeding trial (*n* = 3).

Item	Diets (g/kg)				
	T1	T2	T3	T4	T5
ALB (g/dl)	0.88 ± 0.03^b^	0.78 ± 0.01^a^	1.24 ± 0.06^c^	1.18 ± 0.03^c^	0.92 ± 0.09^b^
ALKP (*μ*/l)	6.25 ± 0.05^a^	7.27 ± 0.21^c^	7.67 ± 0.32^d^	6.34 ± 0.05^a^	6.93 ± 0.06^b^
ALT (*μ*/l)	21.23 ± 0.32^a^	25.93 ± 0.06^b^	25.37 ± 0.2^ab^	25.37 ± 0.32^ab^	27.93 ± 0.2^c^
BUN (mg/dl)	1.17 ± 0.05^a^	1.34 ± 0.04^b^	1.30 ± 0.01^b^	1.61 ± 0.01^c^	2.03 ± 0.06^d^
Ca^2+^ (mg/dl)	6.77 ± 0.23^d^	6.46 ± 0.12^b^	4.53 ± 0.32^a^	6.17 ± 0.12^c^	6.57 ± 0.29^c^
CHOL (g/dl)	25.30 ± 0.26^b^	25.30 ± 0.35^b^	21.77 ± 0.32^a^	25.2 ± 0.35^b^	27.17 ± 0.1^c^
CREA (mg/dl)	0.86 ± 0.03^a^	0.85 ± 0.03^a^	0.85 ± 0.03^a^	0.90 ± 0.02^a^	1.17 ± 0.06^b^
TP (g/dl)	1.13 ± 0.06^a^	1.33 ± 0.06^b^	1.33 ± 0.06^b^	1.73 ± 0.06^d^	1.51 ± 0.01^c^

Note: different superscripts in each row indicate significant differences (*p* < 0.05). Abbreviations: ALB = albumin; TP = total protein; BUN = blood urea nitrogen; CREA = creatine; ALKP = alkaline phosphatase; ALT = alanine aminotransferase; Ca^2+^ = calcium ion; CHOL = cholesterol. Treatment group: T1 (0% BSFL), T2 (6.5% BSFL), T3 (13% BSFL), T4 (19.5% BDFL) and T5 (24.5% BSFL).

**Table 8:** Gut morphology of *B. splendens* after the 60-day feeding trial (*n* = 3).

Item	Intestine region	Diets (g/kg)				
		T1	T2	T3	T4	T5
Villus length (*μ*m)	Anterior	123.7 ± 0.6^a^	140.1 ± 1.1^b^	181.1 ± 1.2^c^	183.6 ± 1^c^	129.3 ± 1.3^a^
	Posterior	106.8 ± 3.1^a^	129.9 ± 1.8^b^	167.9 ± 5.8^c^	162.7 ± 2.9^c^	101.6 ± 1.3^a^
Villus width (*μ*m)	Anterior	68.5 ± 0.4^b^	76.3 ± 1.0^c^	128.0 ± 6.8^d^	129.1 ± 2.5^d^	46.9 ± 2.7^a^
	Posterior	60.6 ± 0.6^b^	67.6 ± 0.7^b^	101.2 ± 2.6^c^	110.3 ± 0.8^d^	36.1 ± 8.5^a^
Crypt depth (*μ*m)	Anterior	41.6 ± 0.9^b^	45.9 ± 0.6^c^	73 ± 0.72^d^	81.03 ± 1.5^e^	32.2 ± 2.7^a^
	Posterior	37.7 ± 0.7^b^	40 ± 0.8^b^	66.3 ± 2.58^c^	68.9 ± 3.17^c^	29.1 ± 1.6^a^

Note: different superscripts in each row indicate significant differences (*p* < 0.05). Treatment group: T1 (0% BSFL), T2 (6.5% BSFL), T3 (13% BSFL), T4 (19.5% BDFL), and T5 (24.5% BSFL).

## Data Availability

The data that support the findings of this study are available on request from the corresponding authors (Zulhisyam Abdul Kari/Noor Khalidah Abdul Hamid).
